# Experimental Investigation on Graphene Oxide/SrCl_2_·6H_2_O Modified CaCl_2_·6H_2_O and the Resulting Thermal Performances

**DOI:** 10.3390/ma11091507

**Published:** 2018-08-22

**Authors:** Zhiyang Jin, Yuanyuan Tian, Xiaoxiao Xu, Hongzhi Cui, Waiching Tang, Yanchun Yun, Guoxing Sun

**Affiliations:** 1 Guangdong Provincial Laboratory of Durability for Marine Civil Engineering, Shenzhen University, Shenzhen 518060, China; jinzhiyang2016@email.szu.edu.cn (Z.J.); 2150150417@email.szu.edu.cn (Y.T.); 2150150414@email.szu.edu.cn (X.X.); 2 School of Architecture and Built Environment, The University of Newcastle, Callaghan, NSW 2308, Australia; patrick.tang@newcastle.edu.au; 3 Baoye Group Company Limited, Shanghai 312030, China; yunyanchun@126.com; 4 Institute of Applied Physics and Materials Engineering, University of Macau, Macau 999078, China; gxsun@umac.mo

**Keywords:** phase change material, supercooling, graphene oxide, SrCl_2_·6H_2_O, CaCl_2_·6H_2_O, thermal performance

## Abstract

Although the inorganic salt hydrate phase change materials (PCMs) such as CaCl_2_·6H_2_O have promising potential for thermal energy storage in building application, the issue of supercooling has restricted their practical application. In this study, graphene oxide (GO) and SrCl_2_·6H_2_O as binary nucleation agents were used to modify CaCl_2_·6H_2_O and reduce its supercooling degree. Compared with pure CaCl_2_·6H_2_O, the incorporation of graphene oxide (GO)/SrCl_2_·6H_2_O reduced the supercooling degree to 0.3 °C significantly. In addition, the supercooling degree of modified CaCl_2_·6H_2_O after 200 thermal cycles was still much lower than that of non-modified CaCl_2_·6H_2_O. From the results of differential scanning calorimetry (DSC), the latent heat value and phase change temperature of the modified CaCl_2_·6H_2_O were 207.88 J/g and 27.6 °C, respectively. Aluminum capsules were used to encapsulate the modified PCM and placed inside the composite wallboard. The thermal performances of the composite wallboard with modified PCM were investigated using infrared thermography. Experimental results showed that the average temperature difference between the top and bottom surfaces of modified CaCl_2_·6H_2_O/wallboard composite after 1 h heating was kept around 15.8 °C, while it was 4.9 °C for the control wallboard. The above test results proved that the modified CaCl_2_·6H_2_O demonstrated good thermal performance and can be used in buildings to maintain thermal comfort.

## 1. Introduction

Inorganic phase change materials (PCMs) generally are non-flammable, readily available, and cheap, but the issues of corrosive to metal and supercooling have restricted their utilization in practical applications. Apparently, an inorganic PCM without the aforesaid issues can be seen as a promising candidate for building application [1,2]. In order to overcome the negative influence caused by supercooling, some scientific mechanisms and measures on inorganic PCMs have been reported. According to the current literature [3] in relation to supercooling reduction, nucleating agents are commonly used to mitigate the supercooling of inorganic PCMs. Some nucleating agents, such as copper foam (for thermal conductivity and matrix), carboxyl methyl cellulose (CMC, thickener), disodium hydrogen phosphate dodecahydrate (DHPD, nucleator), and sodium acetate trihydrate (SAT), were commonly used and studied [4,5], however the supercooling of inorganic PCM is still not completely resolved. 

Recently, due to the rapid development of nanomaterials, some nanomaterials can be used as nucleating agents to stimulate the crystallization of inorganic PCMs and some of them can certainly change the properties of PCMs [4,6]. For instance, some researchers [7,8,9] found that adding graphene (0.02 wt %) could reduce the supercooling degree of pure water. On other studies, Li et al. [10,11] confirmed that SrCl_2_·6H_2_O could reduce the supercooling of inorganic PCM. Therefore, it has been suggested that the usage of graphene oxide (GO) can be greatly reduced with the presence of SrCl_2_·6H_2_O to have the effect on supercooling reduction. Furthermore, the thermal performance of PCMs can be enhanced due to high thermal conductivity of GO. 

In order to enable PCMs to be used in buildings, they need to be encapsulated so as to avoid leakage likeliness. Many methods and measures have been proposed in the literature. For example, Cui et al. [12] and Fu et al. [13,14] respectively introduced interfacial polymerization method and vacuum-impregnation method to envelop PCM to be shape-stabilized PCM. However, the above methods could only store a low content of PCM inside the PCM composite materials. In this study, an aluminum foil capsule was proposed to carry a high volume of PCM in the building envelopes. Owing to its excellent corrosion resistance, the aluminum foil can also minimize the corrosion effects of hydrate salts.

In our previous study, the effect of individual graphene or SrCl_2_·6H_2_O on supercooling of CaCl_2_·6H_2_O had been investigated [15]. In this study, therefore, we proposed the use of GO/SrCl_2_·6H_2_O to modify CaCl_2_·6H_2_O and reduce its supercooling. It is believed that the modified CaCl_2_·6H_2_O composites with high latent heat and supercooling-free would be very attractive for future use in energy-efficient buildings to achieve thermal comfort. In order to avoid deliquescence and metal corrosion, aluminum foil capsules were used to carry the modified CaCl_2_·6H_2_O. The thermal performances of composite wallboard with modified CaCl_2_·6H_2_O were investigated using infrared thermography.

## 2. Materials and Methods 

### 2.1. Materials

In this study, the modified CaCl_2_·6H_2_O composite was prepared from several raw materials. Anhydrous calcium chloride (CaCl_2_) and strontium chloride hexahydrate (SrCl_2_·6H_2_O) with purity >99% were supplied from Guangdong, China, Huada Chemical Co., Ltd. Deionized water was obtained from Guangdong, China, Hugke Water Treatment Equipment Co., Ltd. An industrial-grade graphene oxide nano-sheets (thickness < 5 nm, purity > 99%, size 2–8 µm) with an interlayer distance of 0.824 nm was supplied from Chengdu Organic Chemical Co. Ltd., Chinese Academy of Sciences. Other materials such as polystyrene foam plate (thickness = 45 mm), metal sheet and glass glue were obtained from the local building materials market in Shenzhen, China. The nucleating effects of GO were characterized by Fourier transform infrared (FT-IR, Perkin-Elmer, Waltham, MA, USA) spectroscopy and scanning electron microscopy (SEM, Quanta TM 250 FEG, Hillsboro, OR, USA).

### 2.2. Preparation of Modified CaCl_2_·6H_2_O

To reduce the supercooling degree of CaCl_2_·6H_2_O, GO, and SrCl_2_·6H_2_O were added as binary nucleating agents. The procedures of preparation are as follows: Firstly, 0.02 wt % GO (weight for CaCl_2_·6H_2_O) was initially mixed with 50 g deionized water and followed by sonication for about 30 minutes to ensure better dispersion results. The sonication was carried out by a rod-model ultrasonic machine (JY-92-IIN, Ningbo Xinzhi Biotech Co., Ltd., Ningbo, China, 25 kHz, power: 375 W). Then, the anhydrous CaCl_2_ (50 g) was added to the GO suspension and agitated using a mechanical agitator for a total of 30 min in a water-bath (HH-2, Zhiborui Instrument Manufacturer, Changzhou, China) at a temperature of 40 °C. After ultrasonic dispersion, SrCl_2_·6H_2_O (0.8 wt %) was added and finally the modified PCM composite was prepared.

### 2.3. Thermo-Physical Performance of Modified CaCl_2_·6H_2_O

The thermo-physical performances of modified CaCl_2_·6H_2_O were characterized by differential scanning calorimetry (DSC, DSC-200L, Nanjing Dazhan Electrical Technology Company, Nanjing, China) and T-history method to obtain the latent heat value and supercooling degrees, respectively. The supercooling degree of modified CaCl_2_·6H_2_O under various thermal cycling conditions was studied. The samples were placed in a temperature and humidity programmable chamber (TEMI 300, Dongguan Bell Test Equipment Co., Ltd., Dongguan, China). The thermal cycle started when the sample was heated from 10 to 40 °C in 10 min (at a rate of 3 °C/min) and maintained at 40 °C for 30 min. Then, the temperature was reduced from 40 to 10 °C in 10 min (at a rate of −3 °C/min), and the temperature was held at 10 °C for 30 min, and then the cycle ended. A total of 200 thermal cycles were conducted where the humidity was remained constant for 50 RH %.

### 2.4. Encapsulation of Modified CaCl_2_·6H_2_O

**Figure 1:**
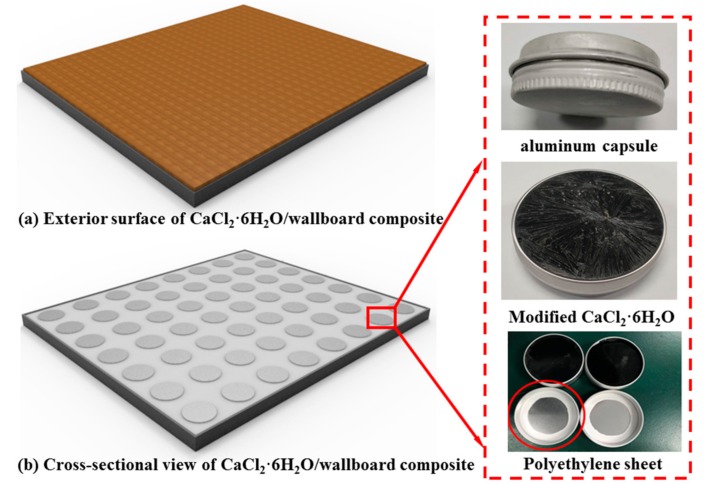
Diagramming of CaCl_2_·6H_2_O/wallboard composite: (**a**) exterior surface of CaCl_2_·6H_2_O/wallboard composite; (**b**) cross-sectional view of CaCl_2_·6H_2_O/wallboard composite.

As shown in Figure 1, there was a thin piece of polyethylene (PE) sheet embedded inside the cap of each aluminum container (Diameter: 52 mm, Height: 20 mm) to prevent leakage. The procedures for encapsulation of modified CaCl_2_·6H_2_O are as follows: Firstly, the modified CaCl_2_·6H_2_O was packaged inside an aluminum capsule and then the capsule was stored in a refrigerator at −20 °C for 2h. Besides, a polypropylene (PP) bag was used to wrap each aluminum container for secondary protection and finally the bag was vacuumed and sealed.

### 2.5. Preparation of Modified CaCl_2_·6H_2_O/Wallboard

The aluminum capsules containing modified CaCl_2_·6H_2_O were used to form modified CaCl_2_·6H_2_O/wallboard composite with a dimension of 650 mm × 550 mm × 30 mm. Each modified CaCl_2_·6H_2_O/woodboard composite consists of three layers as shown in Figure 1a,b. The bottom layer is a thin metal board with a thickness of 4 mm. The metal board was used to resist the gravity of above layers. The layer in middle was a polyethylene sheet (thickness: 21 mm) embedded regularly with 56 aluminum containers wrapped with PP package. Glass glue was used to fill the gap between the capsules and polyethylene layer for better compactness and to avoid unwanted heat transition. The top layer is a thin woodboard with a thickness of 5 mm. A control wallboard without the modified CaCl_2_·6H_2_O was used for comparison. 

### 2.6. Thermo-Regulated Performance of Modified CaCl_2_·6H_2_O

**Figure 2:**
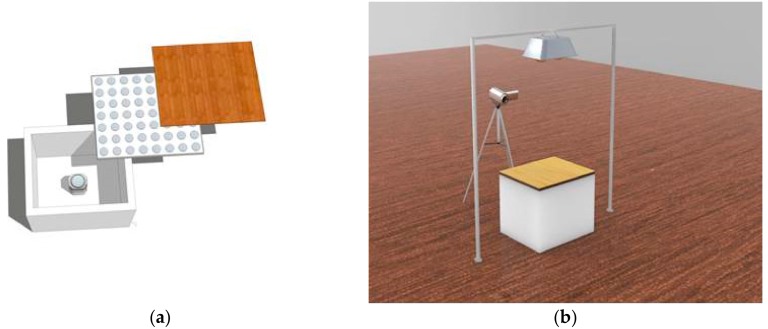
Schematic diagram of testing of thermo-regulated performance: (**a**) room model; (**b**) testing system.

To provide better and distinctive observation of surface temperatures, infrared thermography technique was used to record the temperature variation and heat distribution of the top and bottom surfaces of wallboards after a prolonged heating time. In order to eradicate the environmental impact to the bottom surface temperature, a special room model made of polyethylene was used, as shown in Figure 2. The CaCl_2_·6H_2_O /woodboard was used as roof panel on the room model with a dimension of 650 mm × 550 mm × 550 mm. The room models, except the roof, were all made of polyethylene (thickness: 45 mm). One of the two infrared cameras (Ti450, Fluke, Seattle, WA, USA) was installed at the bottom of room model and responsible for recording the internal (bottom) surface temperature of composite woodboard. Another camera was used to record the external (top) surface temperature at the same time. The heat lamp was placed one meter above the room model and operated for 7 h.

## 3. Results and Discussions

### 3.1. Thermo-Physical Performance of Modified CaCl_2_·6H_2_O

**Figure 3:**
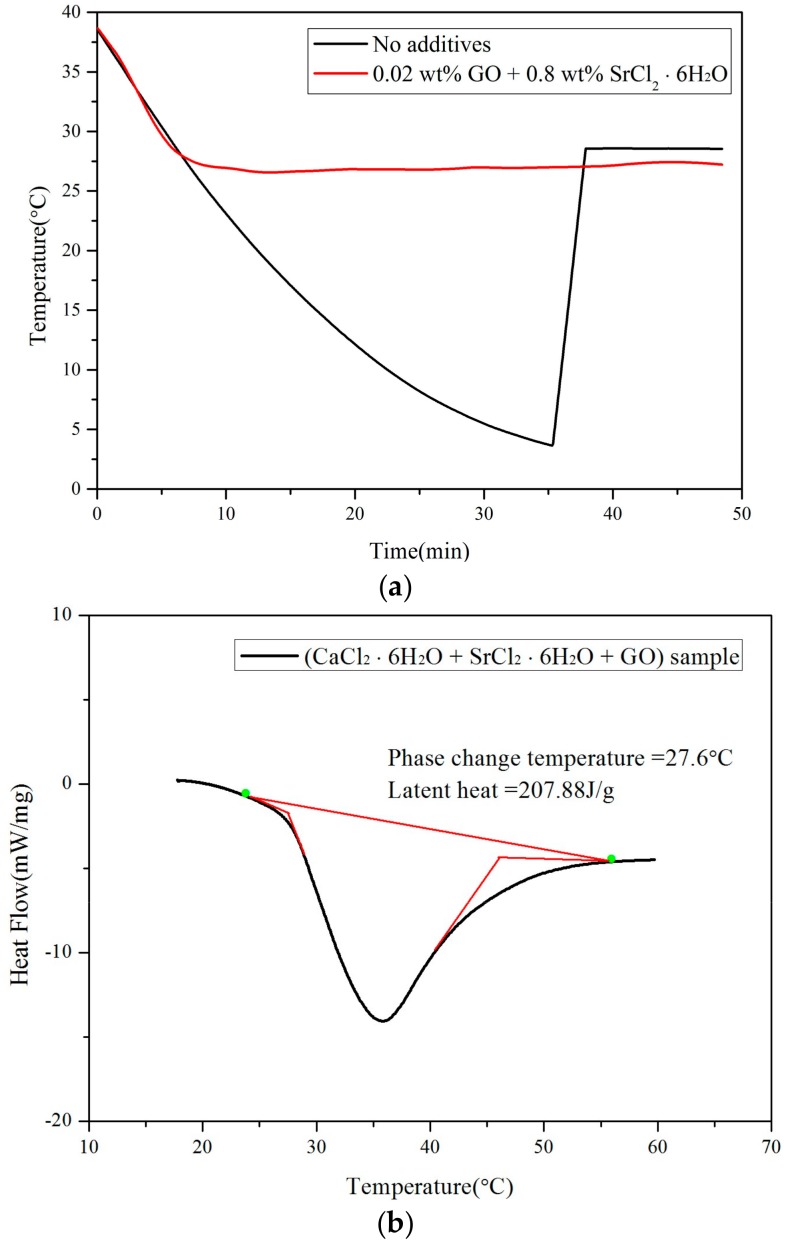
Thermo-physical performance of modified CaCl_2_·6H_2_O: (**a**) curve of supercooling degree; (**b**) differential scanning calorimetry (DSC) curve.

**Figure 4:**
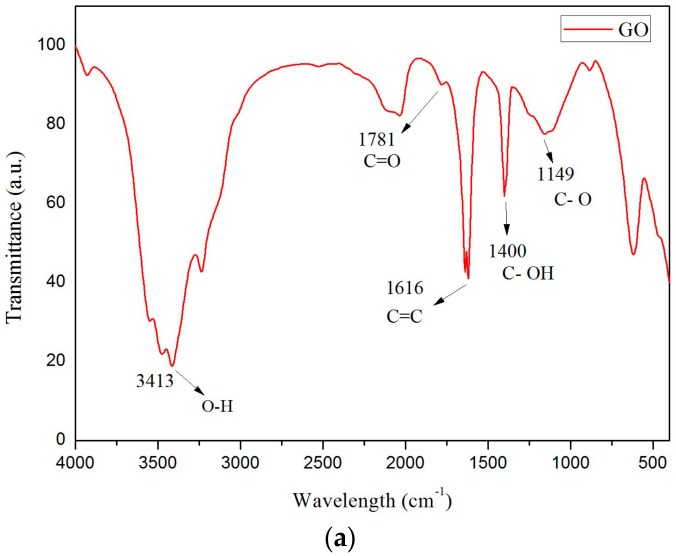

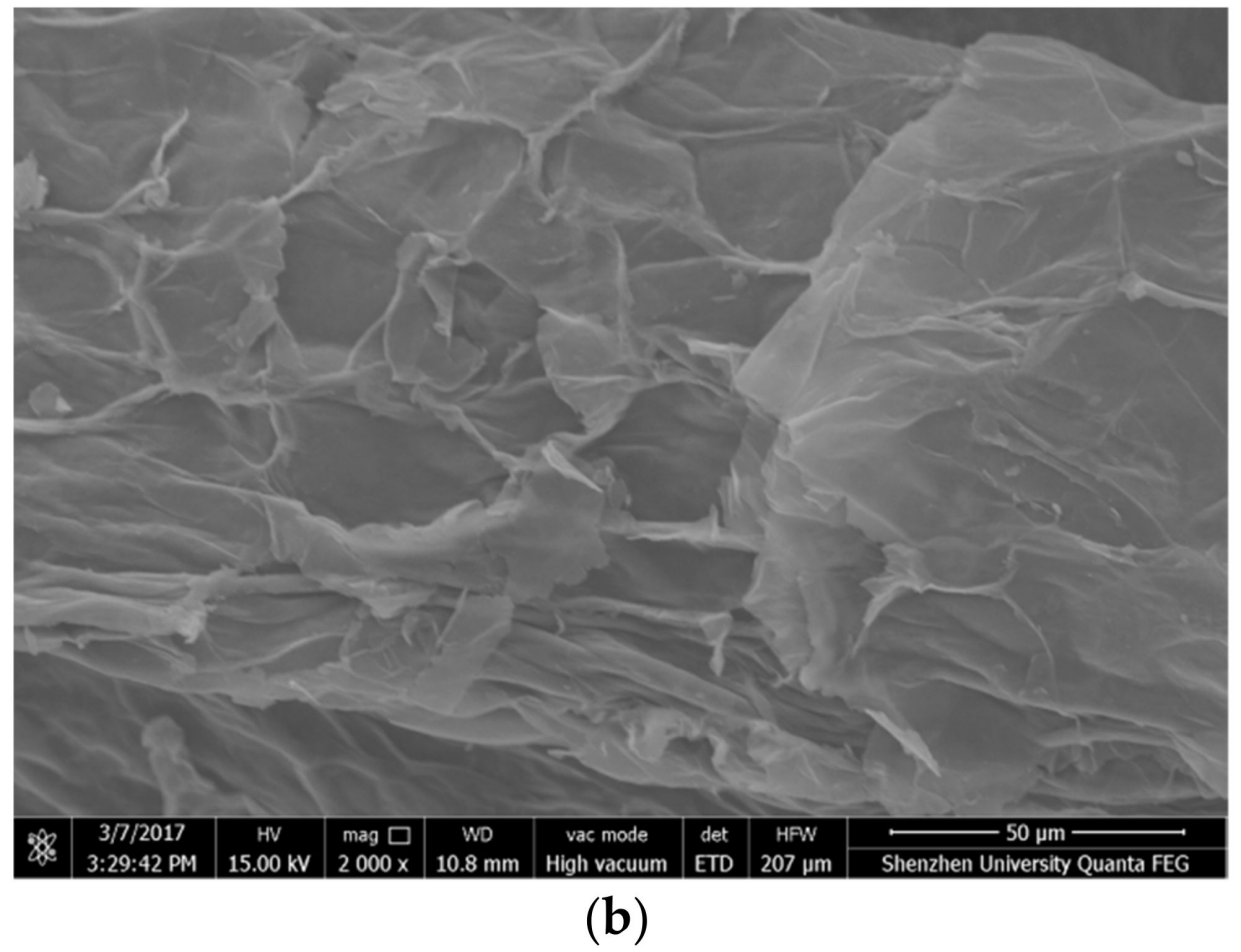
Fourier transform infrared (FT-IR) spectrum (**a**) and SEM image (**b**) of graphene oxide.

The supercooling degree of modified CaCl_2_·6H_2_O is shown in Figure 3a. The cooling curve of the modified CaCl_2_·6H_2_O displays a huge difference compared to the control (without GO and SrCl_2_·6H_2_O). After the modification, the supercooling degree of CaCl_2_·6H_2_O reduced significantly from 25.4 °C to 0.3 °C. Having a reduction of 98.8% supercooling degree, it can be concluded that the binary nucleation agents are super effective to stimulate the crystallization of CaCl_2_·6H_2_O, and supercooling is no longer a concern in the modified PCM. The reduction of supercooling degree can be explained by the presence of oxygen-containing functional groups on the surface of GO which have been recognized as a potential platform in crystal attachment due to better wetting and adsorption [16]. According to the FT-IR results, a powerful absorption at 3413 cm^−1^ due to O–H stretching vibration could be seen in Figure 4a. C=O stretching was also observed at 1781 cm^−1^, and the absorption around 1400 cm^−1^ was attributed to the tertiary C–OH groups. The peak occurred at 1149 cm^−1^ was stretching vibrations of C–O [17,18]. These functional groups indicated the oxidation process of GO was in place. Besides, as shown in Figure 4b, the GO sheet can be characterized as nano-scale thin with large surface area for the attachment of oxygen functional groups. Thus, better dispersion of GO by means of ultra-sonication can effectively modify CaCl_2_·6H_2_O [19]. Besides, Figure 3b shows the DSC results of modified CaCl_2_·6H_2_O. According the provisions of the International Confederation for Thermal Analysis (ICTA) standardization committee, the intersection of the front extension baseline and the maximum frontier slope of the peak represents the melting point, and the area of the peak represents the latent heat. Hence, the latent heat of modified CaCl_2_·6H_2_O was around 207.88 J/g, which is higher than those reported in other studies (170 J/g and 191 J/g) [20]. The phase change temperature was 27.6 °C, which is within the operational temperature and suitable for building applications. 

**Figure 5:**
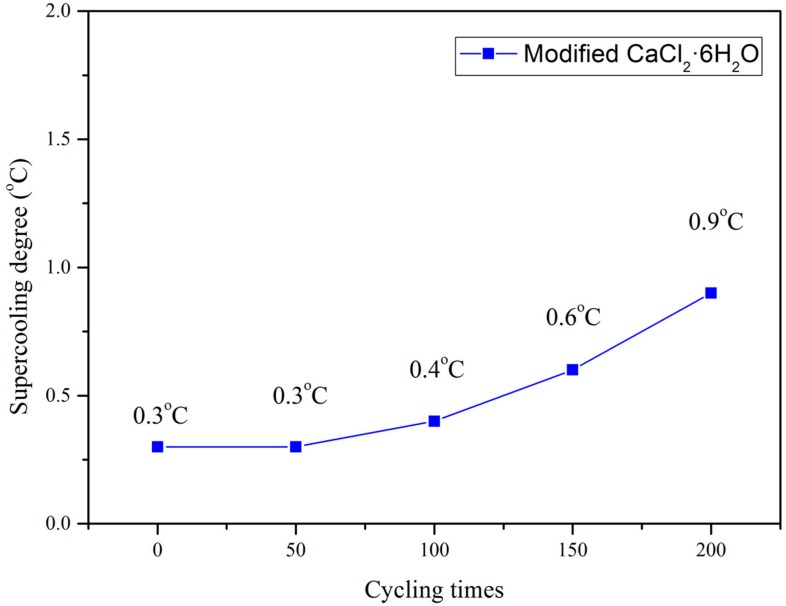
Supercooling degree of modified CaCl_2_·6H_2_O underwent different thermal cycles.

Figure 5 shows the thermal cycle test results of modified CaCl_2_·6H_2_O. From the figure, it can be seen that the supercooling degree slightly increased with increasing thermal cycles, but it was still remained less than 1 °C after 200 thermal cycles. It is well known that supercooling is a major issue which hampers the long-term performance of PCM. In this study, the supercooling degree of modified CaCl_2_·6H_2_O after 200 thermal cycles was still much lower than that of pure CaCl_2_·6H_2_O. Thus, the newly modified CaCl_2_·6H_2_O is more suitable for practical application. Further study about effect of thermal cycles more than 200 times on the supercooling degree of the modified CaCl_2_·6H_2_O will be presented in future publication.

### 3.2. Thermo-Regulated Performance of Modified CaCl_2_·6H_2_O

**Figure 6:**
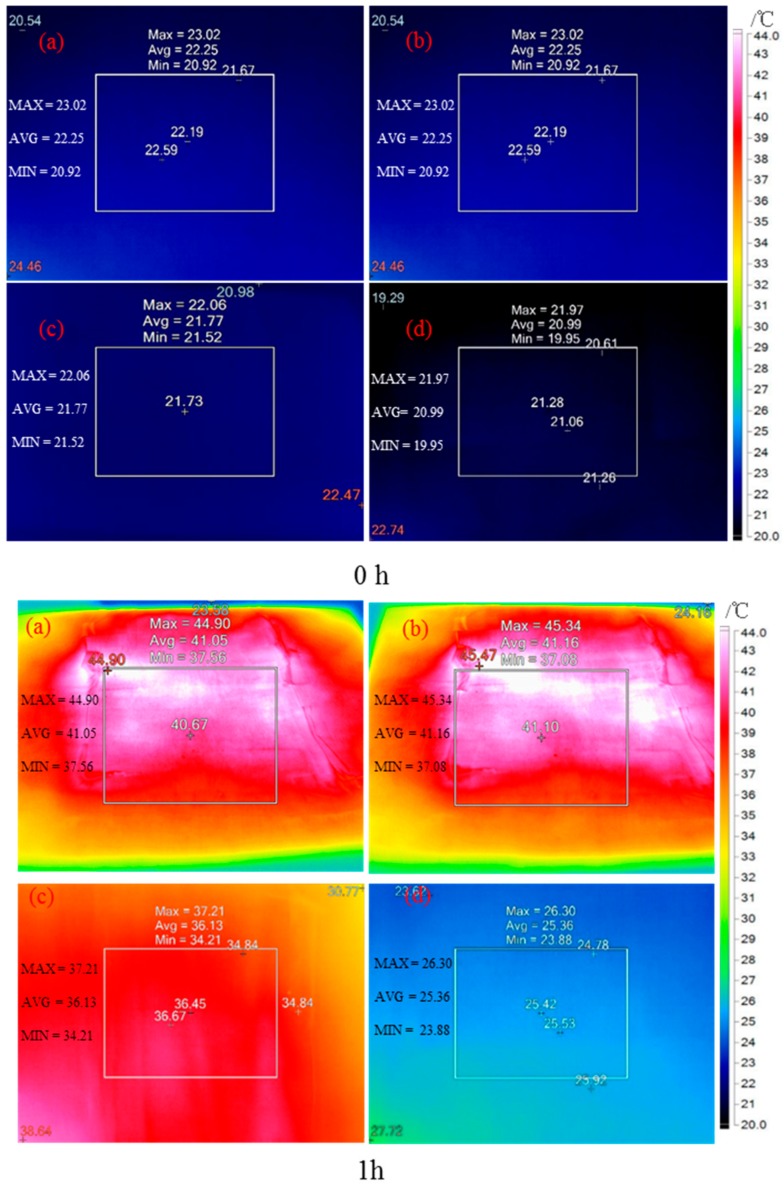

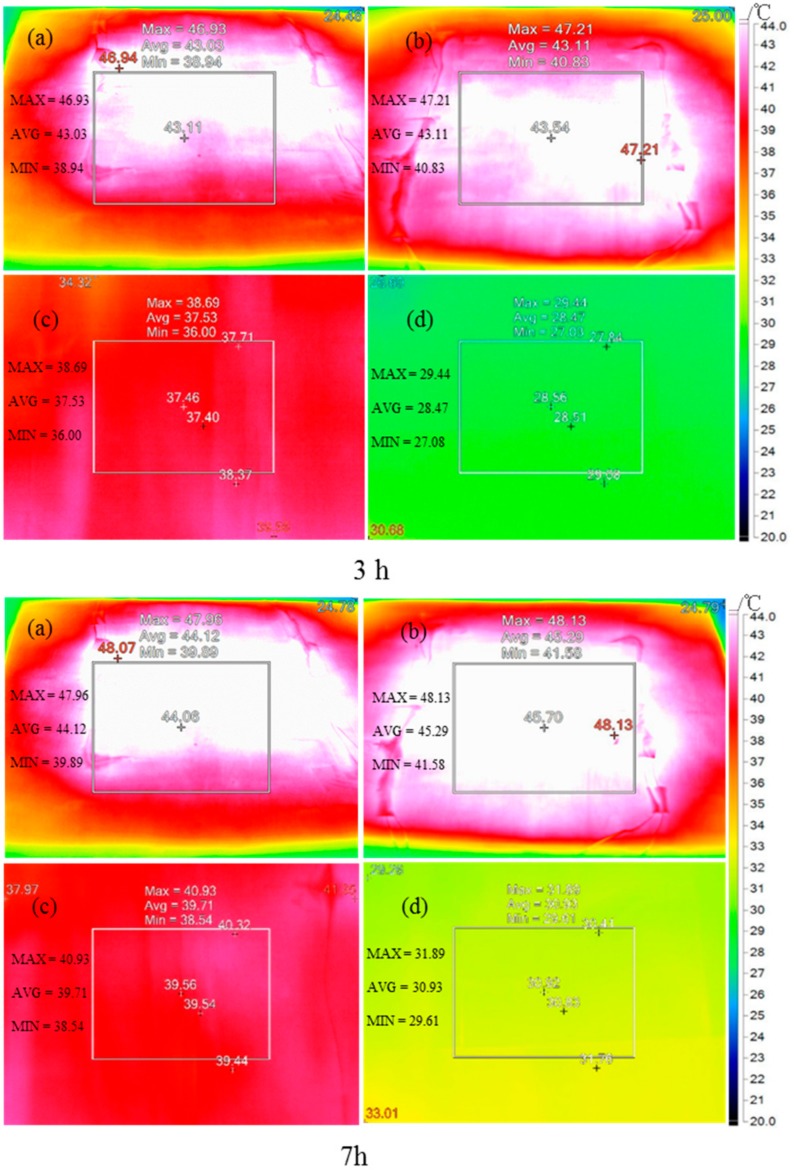
Thermal map of control wallboard: (**a**) top surface and (**c**) bottom surface, and modified CaCl_2_·6H_2_O/wallboard composite: (**b**) top surface and (**d**) bottom surface.

Figure 6 display the top and bottom surface temperature distribution of the control and modified CaCl_2_·6H_2_O /wallboard composites, respectively. Table 1 shows the average temperature of two kinds of wallboard. The infrared thermography results show that the bottom surface of control wallboard got heated up quickly due to the direct heat transition from top to the bottom. However, the modified CaCl_2_·6H_2_O/wallboard composite successfully prevented the rapid heat transition from the top wooden board to the bottom metal board, thanks to the high latent heat capacity of modified CaCl_2_·6H_2_O. After heating for 1 h, the highest average temperature difference between the top and bottom surfaces of control wallboard was around 4.9 °C. In contrast, the average temperature difference in case of modified CaCl_2_·6H_2_O/wallboard composite was 15.8 °C. After 3 and 7 h continuous heating, the function of modified CaCl_2_·6H_2_O layer in wallboard still remained effective and the temperature difference between the top and bottom layers were maintained at around 14.6 °C and 14.4 °C, respectively. Compared with the control wallboard, the modified CaCl_2_·6H_2_O/wallboard composite showed its strong resistance to transfer of excessive heat from top surface to the bottom surface and demonstrated a great potential for use in building envelope to regulate indoor temperature. After 7 h of prolonged heating, the bottom surface temperature of modified CaCl_2_·6H_2_O/wallboard composite exceeded the melting temperature of PCM (27.6 °C) which represented its capacity loss for energy, and 7 h working time is deemed as the effective service time of modified CaCl_2_·6H_2_O. 

## 4. Conclusions

Based on the above, the following conclusions can be drawn:

(1) In this study, graphene and SrCl_2_·6H_2_O were utilized as nano nucleating agents and successfully reduced the supercooling of pure CaCl_2_·6H_2_O to around 0.3 °C which was much lower than that of the original pure CaCl_2_·6H_2_O (25.4 °C). The latent heat value and phase change temperature of modified CaCl_2_·6H_2_O were 207.88 J/g and 27.6 °C, respectively.
(2) The supercooling degree of modified CaCl_2_·6H_2_O after 200 thermal cycles was still much lower than that of non-modified PCM.
(3) Aluminum capsules wrapped with polypropylene bag can effectively prevent leakage and deliquescence of PCM.
(4) The infrared thermography showed that the temperature difference between the top and bottom layers of modified CaCl_2_·6H_2_O/wallboard composite was 15.8 °C after heating for one hour, while it was 4.9 °C for control wallboard. The results further demonstrated the excellent thermal energy storage and thermal-regulated capacity of modified CaCl_2_·6H_2_O.

## Figures and Tables

**Table 1:** Average temperature of two kinds of wallboard.

No.	T_average_				
	Surface	0	1 h	3 h	7 h
Control group	Top	22.3	41.1	43.0	44.1
	Bottom	21.8	36.1	37.5	39.7
CaCl_2_·6H_2_O/woodboard	Top	22.3	41.2	43.1	45.3
	Bottom	21.0	25.4	28.5	30.9
